# How and why do doctors communicate diagnostic uncertainty: An experimental vignette study

**DOI:** 10.1111/hex.13957

**Published:** 2024-01-09

**Authors:** Caitríona Cox, Thea Hatfield, Zoë Fritz

**Affiliations:** ^1^ The Healthcare Improvement Studies (THIS) Institute University of Cambridge Cambridge UK

**Keywords:** communication, diagnostic uncertainty, doctor–patient relationship, ethics, safety‐netting

## Abstract

**Background:**

Diagnostic uncertainty is common, but its communication to patients is under‐explored. This study aimed to (1) characterise variation in doctors' communication of diagnostic uncertainty and (2) explore why variation occurred.

**Methods:**

Four written vignettes of clinical scenarios involving diagnostic uncertainty were developed. Doctors were recruited from five hospitals until theoretical saturation was reached (*n* = 36). Participants read vignettes in a randomised order, and were asked to discuss the diagnosis/plan with an online interviewer, as they would with a ‘typical patient’. Semi‐structured interviews explored reasons for communication choices. Interview transcripts were coded; quantitative and qualitative (thematic) analyses were undertaken.

**Results:**

There was marked variation in doctors' communication: in their discussion about differential diagnoses, their reference to the level of uncertainty in diagnoses/investigations and their acknowledgement of diagnostic uncertainty when safety‐netting. Implicit expressions of uncertainty were more common than explicit. Participants expressed both different communication goals (including reducing patient anxiety, building trust, empowering patients and protecting against diagnostic errors) and different perspectives on how to achieve these goals. Training in diagnostic uncertainty communication is rare, but many felt it would be useful.

**Conclusions:**

Significant variation in diagnostic uncertainty communication exists, even in a controlled setting. Differing communication goals—often grounded in conflicting ethical principles, for example, respect for autonomy versus nonmaleficence—and differing ideas on how to prioritise and achieve them may underlie this. The variation in communication behaviours observed has important implications for patient safety and health inequalities. Patient‐focused research is required to guide practice.

**Patient or Public Contribution:**

In the design stage of the study, two patient and public involvement groups (consisting of members of the public of a range of ages and backgrounds) were consulted to gain an understanding of patient perspectives on the concept of communicating diagnostic uncertainty. Their feedback informed the formulations of the research questions and the choice of vignettes used.

## 1 INTRODUCTION

### 1.1 Diagnosis, uncertainty and disclosure

**Figure 1:**
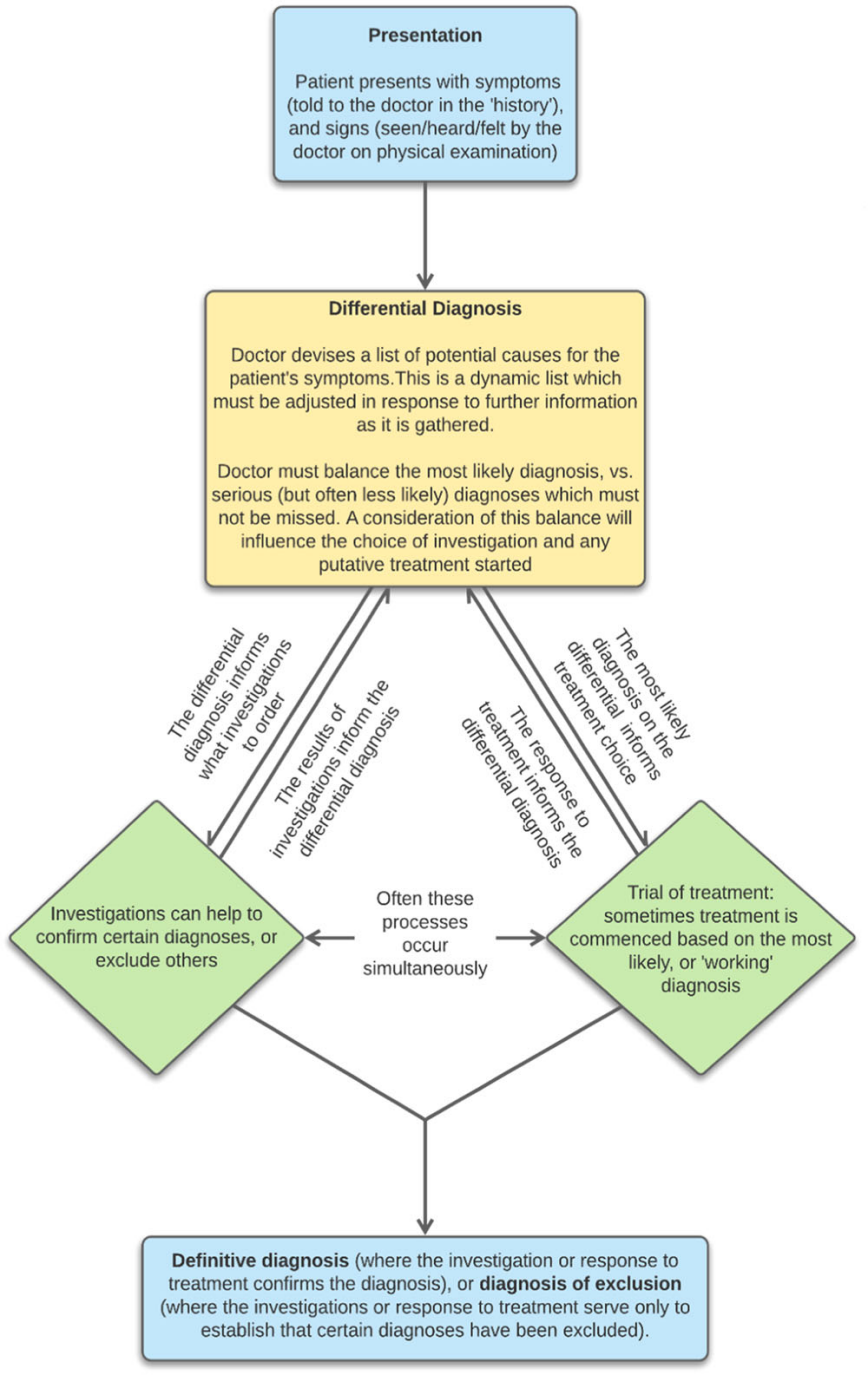
Overview of uncertainty in the diagnostic process.

Diagnosis is not a single event, but a complex and collaborative process with several phases. This process is nonlinear and dynamic, as is the uncertainty within it.^1^, ^2^ Figure 1 (developed using a combination of the authors' clinical experience and review of relevant literature)^1^, ^2^, ^3^, ^4^, ^5^, ^6^ provides an overview of the diagnostic process.

Given its ubiquity, doctors must learn to manage diagnostic uncertainty.^7^, ^8^, ^9^ ‘Managing’ uncertainty is not, however, straightforward: various strategies may be employed by physicians, as captured by Han et al.^10^ These strategies include ‘ignorance‐focused’ strategies (e.g., requesting further investigations), which aim to reduce uncertainty and ‘relationship‐focused’ strategies (e.g., communicating uncertainty to patients), which do not aim to reduce uncertainty but rather mitigate its potential adverse effects.

The communication of uncertainty within medicine has been examined in various contexts.^11^, ^12^, ^13^ Its study is, however, complicated by a lack of consensus on the definition or conceptual model. Han et al.'s^14^ ‘taxonomy of uncertainty’ delineates the source, issue and locus of uncertainty. We focus on the communication of *diagnostic* uncertainty, a topic noted to be particularly underexplored^15^, ^16^; as Meyer et al.^17^ conclude, ‘Little is known about how physicians … communicate diagnostic uncertainty to patients … despite theoretical work exploring medical/physician uncertainty’. Although there is overlap between issues relating to the communication of diagnostic uncertainty and other types of medical uncertainty, to view all uncertainty communication as homogenous would be an oversimplification. Given the centrality of diagnosis to the clinical encounter,^1^, ^18^ patients may respond differently to discussions about uncertainty in diagnosis compared with, for example, prognosis.

There have been calls for open communication of diagnostic uncertainty, from both researchers^2^, ^19^, ^20^ and regulatory bodies.^21^ These recommendations are not particularly evidence‐based: a review suggested that more systemic empirical research is required to substantiate the reasoning for such communication.^20^ This study begins to address this by exploring both *how* and *why* UK doctors currently communicate diagnostic uncertainty to patients.

### 1.2 Ethical and legal background

Medical students are primarily taught ethics through the prism of the ‘four pillars’: autonomy, nonmaleficence, beneficence and Justice.^22^ Discussions regarding information disclosure to patients are often framed in these terms, the ethical tension cited as arising from the need to balance competing ethical principles. Ethical analyses commonly consider how a doctor must balance respect for autonomy (by empowering the patient with information about their condition) with the prevention of harm (from overwhelming the patient with ‘too much’ information, or from distressing information).^23^

The doctor's right to not disclose information if there is reasonable belief that it will result in serious psychological harm to the patient is known as ‘therapeutic privilege’.^24^, ^25^ Whether therapeutic privilege should be extended to diagnostic disclosure has been considered, but there is no clear consensus.^26^ Although doctors may sometimes choose not to share diagnostic uncertainty due to concerns that it may cause harm by causing anxiety, recent professional guidance in the UK states that ‘[i]f you are uncertain about the diagnosis … you should explain this to the patient’.^21^

Legal researchers have contemplated whether doctors have a duty to disclose diagnostic uncertainty.^5^ Case law provides differing perspectives. In the United States, Jandre v. Physicians Insurance Company of Wisconsin (2012) established that a physician could have a duty to disclose diagnostic uncertainty under the ‘reasonable patient' standard.^5^ In the United Kingdom, Montgomery versus Lanarkshire (2015) established that doctors are legally required to disclose ‘material risks’ associated with different treatment options,^5^ but it is unclear if this duty extends to disclosing the differential diagnosis or diagnostic uncertainty.^27^

In summary, the extent to which doctors should communicate diagnostic uncertainty to patients has been recently considered by both legal and ethical scholars. The empirical data presented in this study should be considered within the context of this changing legal and ethical landscape.

### 1.3 What is currently known about diagnostic uncertainty communication, and what does this study add?

Two systematic reviews explored the communication of diagnostic uncertainty in primary^28^ and secondary care.^29^ They revealed a paucity of literature and, echoing the study of uncertainty writ‐large, inconsistencies in definition/measurement. Much of the existing research exploring uncertainty communication has used interviews with doctors; the few studies involving observation of doctor–patient interactions produced conflicting results. Some suggest that diagnostic uncertainty is infrequently communicated,^30^, ^31^ while others observed it more commonly.^1^, ^32^, ^33^ A 2022 study explored how US residents communicated with standardised patients in scenarios without a clear diagnosis: 28% did not discuss diagnostic uncertainty at all, and those who did varied in their use of explicit and implicit language.^34^

Overall, although the communication of uncertainty has been studied, the communication of *diagnostic* uncertainty remains under‐researched, and existing research has produced conflicting results. The present study uses vignettes—hypothetical yet realistic scenarios—to specifically address the research gap regarding how doctors communicate *diagnostic* uncertainty in a UK secondary care context.

Given the changing legal and ethical landscape of diagnosis, and the paucity of research on the communication of diagnostic uncertainty, this study aimed to (1) characterise variation in doctors' communication of diagnostic uncertainty, and (2) explore why variation occurred.

## 2 METHODS

### 2.1 Study design

Participants took part via the Thiscovery platform, an online research platform developed by THIS Institute (University of Cambridge).

Participants read four clinical vignettes (presented in random order to mitigate order effect), each involving significant diagnostic uncertainty. After each vignette, they were asked to tell an interviewer exactly what they would tell a ‘typical patient’. A short semi‐structured interview followed, in which participants were asked to explain why they chose to communicate as they did; what teaching they had received in communicating diagnostic uncertainty and about the realism of the vignettes (semi‐structured interview guide in Appendix SA).

### 2.2 Development of vignettes

Vignettes were developed using the clinical expertise of the authors, established clinical guidelines and expert input from consultants in relevant specialties.^35^, ^36^ We selected common medical presentations to increase participants' ability to recount how they would typically communicate. We developed multiple vignettes to investigate diagnostic uncertainty across a range of clinical scenarios; the vignettes differ from one another in terms of the exact clinical details (e.g., the presentation and the investigation results) but all depict common clinical scenarios encountered in internal medicine (in outpatient vs. inpatient settings), all involving a degree of diagnostic uncertainty. In all the scenarios, the precise diagnosis is not 100% certain, with investigation results ruling certain diagnoses out but not providing an exact cause for the patient's symptoms. See Table 1 for a summary of the vignettes; full copies are in Appendix SB.

**Table 1:** Summary of the vignettes.

Vignette number	Summary of case	Key elements of diagnostic uncertainty
1	40‐year‐old man with 3 years of intermittent abdominal pain, bloating and diarrhoea. No ‘red flag’ symptoms for cancer. Past medical history of migraines and depression. No recent travel and no relevant family history. Normal examination, negative FIT (looked for blood in stool) and negative stool cultures. All tests, including FBC, LFT, thyroid function tests, coeliac serology, faecal elastase and faecal calprotectin, are normal. You believe IBS is the most likely cause of his symptoms.	The exact diagnosis here is not 100% certain—although IBS is the most likely diagnosis, there is no definitive test to confirm this. Without colonoscopy **±** biopsies, there is still a (very) small chance that this could be IBD or even a colorectal malignancy. Most doctors would agree that the chances of these alternate diagnoses are so low that the risks of doing further more invasive tests (such as a colonoscopy) outweigh the benefits.
2	75‐year‐old lady with a background of rheumatoid arthritis, for which she takes methotrexate. She has been more tired than normal in the last few months, with some mild lower back pain. She has not lost weight and nor has she had any night sweats. Recent blood taken as part of her methotrexate monitoring show mild normocytic anaemia (Hb 105), with normal U&Es and LFTs. You feel this is most likely anaemia of chronic disease, but you want to order some further investigations to rule out more serious conditions: iron studies, B12 and folate, LDH, bone profile, a blood film and myeloma screen.	At this stage, the differential diagnosis is very broad—many things could be causing this patient's anaemia, ranging from the more common benign illnesses (e.g., iron or vitamin deficiencies, drug side effects) to the less likely, but more sinister causes (e.g., myeloma, a type of blood cancer). Without these further investigations, we can suggest which of these diagnoses are more or less likely, but we cannot know what the cause of the anaemia is.
3	45‐year‐old man with no past medical history, who presents with central chest pain which came on with mild exertion and lasted 30 min. Normally he cycles 10 miles/day and has never had chest pain before. His maternal uncle died of a myocardial infarction at age 70, but he has no other cardiac risk factors. His examination is normal. CXR, ECG, D‐dimer and serial troponins are all normal. You plan to discharge him with no further follow‐up.	The investigations are all very reassuring and have essentially excluded serious pathologies, such as pneumothorax, pulmonary embolus or myocardial infarction. The cause for the chest pain is not clear—it may be something benign, such as acid reflux or a muscular strain, but this is uncertain. There is a small chance that this is the first presentation of angina, although this is less likely given the patient's lack of risk factors and the fact that he cycles regularly and has never had such pain before.
4	30‐year‐old man with no past medical history who presented to A&E with a severe headache, which came on at rest over a period of approximately 10 min. No associated loss of consciousness, neck pain, rash, photophobia or vomiting. His examination and observations are normal, as are his routine blood tests. He has a CT head within 3 h of headache onset, which is reported by a neuroradiologist as normal. His headache has improved with paracetamol and is now a dull 3/10 severity. You are going to discharge him without a LP.	The normal examination/observations, blood tests and CT scan have essentially ruled out meningitis or a lesion inside the brain (such as a brain tumour). An important diagnosis to consider is a subarachnoid haemorrhage (SAH). Traditionally, a CT was not considered sensitive enough to rule out such a bleed, so if there was a sufficient degree of suspicion patients would go on to have an LP (which is more sensitive at detecting a small bleed). NICE guidance recommends that if the CT scan is done within 6 h of headache onset it can be used to exclude an SAH. For this patient then, we cannot rule out an SAH with 100% certainty, but the risks of doing an LP most likely outweigh the benefits. We do not have a clear cause for the headache—it may be a migraine, but this is uncertain.

Abbreviations: CT, computed tomography; CXR, chest X‐ray; ECG, electrocardiogram; FBC, full blood count; FIT, faecal immunochemical test; IBD, inflammatory bowel disease; IBS, irritable bowel syndrome; LDH, lactate dehydrogenase; LFT, liver function test; LP, lumbar puncture.

We pilot‐tested the vignettes with 12 doctors (from various specialties and grades). Changes were made iteratively in response to feedback on vignette realism, readability and clarity; pilot‐testing continued until minimal changes were suggested. We asked about reasons for communicating (or not communicating) certain information; answers informed the development of the postvignette questions.

### 2.3 Participants and recruitment

We used NHS trust emailing lists to recruit from five hospitals, varying in location and size. Doctors who had worked in general internal medicine for (at least) three of the last 12 months were eligible and were paid a £20 online voucher for participation. To avoid priming participants, the participant information sheet described the study as ‘investigating how different doctors communicate with patients differently’, rather than explicitly mentioning diagnostic uncertainty.

Following the concept of information power,^37^ we planned for an approximate sample size of *n* = 50, intending to adapt according to emerging findings.

### 2.4 Data collection and analysis

Data were collected from 1 February to 18 March 2022. Online interviews were undertaken by C. C., T. H. and Z. F. They were audio‐recorded and transcribed verbatim. NVivo 12 Pro software and Microsoft Excel were used for analysis.

#### 2.4.1 Content analysis of doctor responses to the vignettes

After reading each vignette, doctors were asked to tell an interviewer exactly what they would tell a typical patient. We analysed these responses using a content analysis approach,^38^ utilising both deductive and inductive coding. Initial coding categories describing specific communication behaviours were developed a priori (based on pilot interview data, literature review and the authors' clinical experience); this initial codebook was intentionally broad, designed to capture all topics that doctors might cover in discussing each of the clinical scenarios. In keeping with our research questions, we emphasised information relating to diagnoses and associated certainty/uncertainty.

T. H. and C. C. independently coded the first three transcripts using this initial codebook, adding to it via inductive coding. The whole research team reviewed and discussed any inconsistencies to finalise a coding framework (see Appendix SC for a copy of the codebook). T. H. and C. C. then coded all doctors' answers using this codebook and quantified the frequency of communication on various topics. We recorded the count data of each code, analysing the resulting quantitative data with simple descriptive statistics. We acknowledge that quantitative results should be interpreted with caution given the relatively small sample size.

#### 2.4.2 Thematic analysis of follow‐up semi‐structured interview

The responses to the semi‐structured follow‐up interview were analysed using the constant comparative method.^39^ Using an iterative, open coding approach, we developed codes to capture the main themes interpreted from the data; we compared data from different participants to highlight similarities and differences, considering possible reasons for these. T. H. and C. C. initially independently coded the first three interview transcripts before the whole research team met to review these codes and determine the ongoing coding approach. C. C. and T. H. then read and coded the rest of the transcripts, regularly independently coding the same transcript and meeting to discuss discrepancies. Higher‐level themes were identified and compared with existing literature to highlight trends and gaps.

## 3 RESULTS

We reached theoretical saturation after 36 interviews (Table 2) and therefore closed the study.

**Table 2:** Participant details.

Participant characteristic	*N* (%)
Grade	
Consultant	10 (27.8)
Registrar or equivalent	10 (27.8)
Core trainee or equivalent	9 (25.0)
Foundation doctor	7 (19.4)
Other	0 (0)
Sex	
Female	18 (50)
Male	18 (50)
Prefer not to say	0 (0)
Ethnicity	
White	19 (52.8)
Mixed/multiple ethnic groups	2 (5.6)
Asian/Asian British	15 (41.7)
Black/African/Caribbean	0 (0)
Arab	0 (0)
Other	0 (0)
Prefer not to say	0 (0)
NHS trust	
Hospital A (teaching hospital)	13 (36.1)
Hospital B (district general hospital)	10 (27.8)
Hospital C (dstrict general hospital)	8 (22.2)
Hospital D (teaching hospital)	3 (8.3)
Hospital E (teaching hospital)	2 (5.6)

We first present the results of our content analysis (exploring *what* information doctors communicated in response to the vignettes), followed by the results of the thematic analysis of the follow‐up interviews (exploring *why* doctors communicated as they did).

### 3.1 How do doctors communicate when faced with diagnostic uncertainty?

Figure 2 shows the quantitative results (the percentage who discussed each code for each of the vignettes).

Figure 2Percentage of the participants who discussed codes in each vignette. CT, computed tomography; ddx, differential diagnosis; dx diagnosis; hx, history; IBS, irritable bowel syndrome; Ix investigation; LP, lumbar puncture; SAH, subarachnoid haemorrhage.

**Figure:**
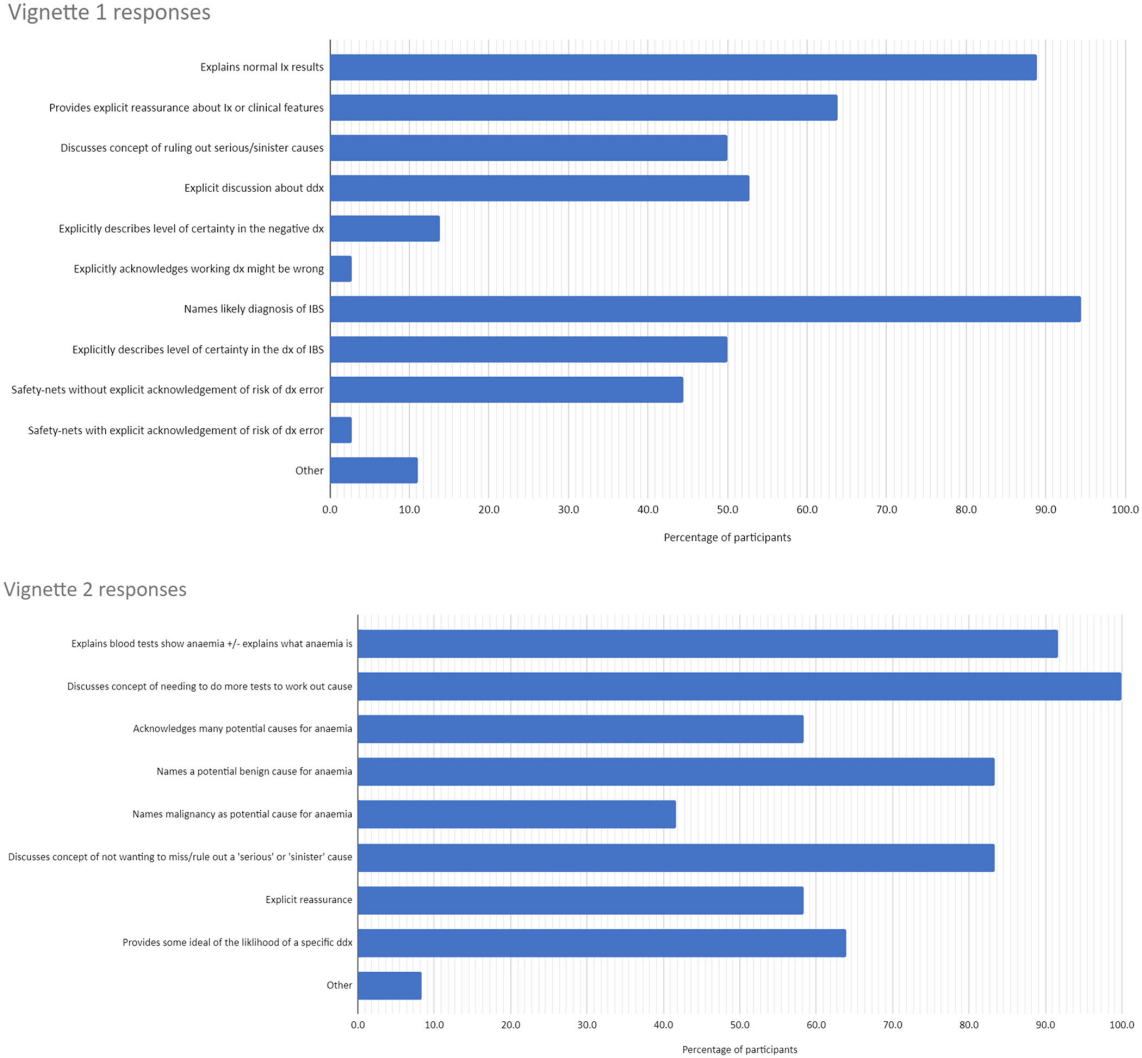

**Figure:**
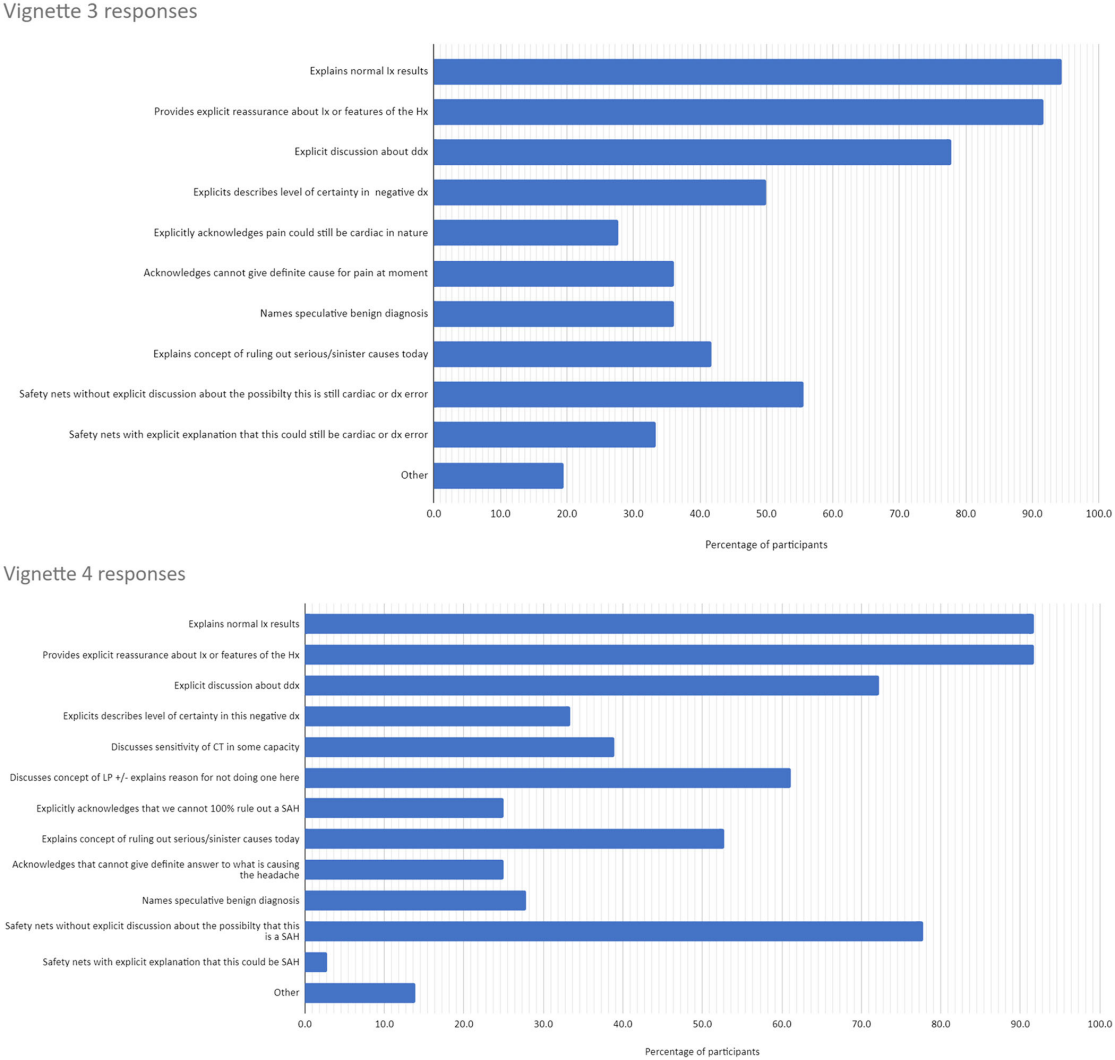

Across all vignettes, implicit expressions of uncertainty were more common than explicit: participants rarely explicitly stated the working diagnosis might be incorrect, or openly acknowledged that they did not have a definite diagnosis. Although these commonalities were present, there was significant variation in what information was communicated, to the ‘typical’ patient. We present the main areas of variation below, with illustrative quotations in Table 3.

**Table 3:** Summary of the main variation in what was communicated about diagnostic uncertainty.

Topic	Communication behaviours observed	Illustrative quotations
Discussion about the differential diagnosis.	Full discussion of the diagnoses which were being investigated for	‘So the possible things that can be going on would be things like inflammatory bowel disease, such as Crohn's or ulcerative colitis… Sometimes we worry about whether there could be any problem with the absorption due to your pancreas not producing particular enzymes’. ‘[W]hat I'm talking about are things like heart attacks … blood clots on the lungs; something called a pulmonary embolism, and other serious causes like a collapsed lung or pneumonia’.
	Only mentions the most likely diagnosis, without discussing other differentials	‘Thankfully, we have not found anything sinister on you … the most likely diagnosis, at the present moment, would be what you call IBS, that's irritable bowel syndrome’.
	Acknowledgement of malignancy as a potential diagnosis	‘There are other things that can cause an anaemia and these things are nastier. So we're talking about certain types of blood cancers’.
	Only refers to benign diagnoses	‘Now one of your blood tests has shown a slightly low haemoglobin count. Now this is something that could be caused by a lot of different reasons. So, just having the rheumatoid arthritis, as you've got, can cause a slightly low haemoglobin count, that's very common. And also one of the medications you're on can sometimes cause a bit of a low haemoglobin count. So, you've got some good reasons to have this, and so it may just be related to that and it wouldn't be anything concerning’.
The level of certainty in the suggested diagnosis or in diagnoses that had been excluded.	Use of qualifiers to give an indication of the level of certainty	‘I think given your background and the symptoms you're describing … the most likely diagnosis is irritable bowel syndrome’.
	Discussion about diagnoses with no reference to level of certainty	‘The diagnoses I've come to for your symptoms is of irritable bowel syndrome’. ‘[W]e've ruled out everything sinister for the moment’.
	Explicit acknowledgement that working diagnosis is not 100% certain	‘It's likely to be something called irritable bowel syndrome. And obviously, I can't say that this is the diagnosis altogether, but it's something that we need to monitor’.
The level of certainty in investigations.	Implies that investigations definitely rule in or rule out diagnoses	‘[W]e would like to rule out a bleed inside the brain, which we have done by doing a CT scan of your head’.
	Discusses the level of certainty in investigations	‘[B]ecause d‐dimer is one of the blood tests which has a very high negative predictive value, in the sense if it's a negative, it's very unlikely to be a lung clot’.
	Explicit acknowledgement that investigations are not 100% certain	‘[T]he CT scan isn't 100 per cent at ruling out one of these bleeds on the brain called a subarachnoid haemorrhage’.
Acknowledgement that a clear cause for symptoms could not be provided.	Explicit acknowledgement that diagnosis is not clear	‘I can't tell you exactly what your chest pain was’ ‘In this kind of situation where we don't have a very clear cause for your headache … it can be a bit difficult to fully explain your symptoms’.
	Suggestion of a speculative benign diagnosis.	‘[I]t's likely a musculoskeletal sort of pain, so something from the musculature rather than from your heart itself’.
Safety‐netting communication.	Safety‐netting without acknowledgement of risk of diagnostic error.	‘[I]f the headache returns, any neurological signs, any neck stiffness or anything like that, you should come back into hospital’.
	Safety‐netting with acknowledgement of risk of diagnostic error.	‘[I]f this headache suddenly gets worse or you develop anymore symptoms … you need to come back to us again and we need to revisit the whole situation just to make sure we haven't missed anything’.

#### 3.1.1 The differential diagnosis

Discussion about the differential diagnosis was common but not universal. Although most participants referred to a range of potential diagnoses, others mentioned only a single diagnosis (typically the most likely/most important to exclude diagnosis). For example, in the change of bowel habit (CoBH) vignette, 53% of the participants discussed differential diagnoses that had been considered, while 47% only mentioned the most likely diagnosis (irritable bowel syndrome [IBS]).

Discussion about benign differential diagnoses was more common than malignant. In the anaemia vignette, 83% of the participants named a potential benign cause, but only 42% explicitly mentioned malignancy (despite myeloma being an important differential that was being investigated for).

#### 3.1.2 Level of certainty in *diagnoses*

Approximately half of the participants indicated how certain they were in the diagnoses they suggested, through phrases such as ‘most likely’ or ‘probable’. Others communicated in a more binary fashion, excluding certain diagnoses and confirming others without indicating the relative certainties. In the CoBH vignette half of the participants did not give any indication of uncertainty, instead presenting IBS as a firm definite diagnosis; just one participant explicitly stated that the working diagnosis of IBS might be wrong.

#### 3.1.3 Level of certainty in *investigations*

Communication about the level of certainty associated with investigations was uncommon: a small number of participants mentioned how sensitive tests were at detecting abnormalities, but most explained the normal investigations without mentioning the level of certainty afforded by the results. In the headache vignette, just 39% of the participants referred to the concept of sensitivity when discussing the computed tomography (CT) scan, and only one participant explicitly stated that the scan was not 100% sensitive in ruling out a subarachnoid haemorrhage.

#### 3.1.4 Acknowledgement that a definite cause for symptoms could not be provided

In the chest pain and headache vignettes, there was no clear cause for the symptoms. Only a minority of participants explicitly acknowledged this: in the chest pain vignette, 36% acknowledged that they could not explain the pain, and in the headache vignette, it was just 25%. A similar percentage (36% and 28% in chest pain and headache vignettes) offered a speculative benign diagnosis to explain the cause for symptoms (e.g., musculoskeletal pain or migraine).

#### 3.1.5 Safety‐netting

Safety‐netting—providing instructions about when to return to seek further medical advice—was common across all vignettes. Most safety‐netting was done without any explicit reference to the risk of diagnostic error: participants explained what symptoms to look out for, but did not explain that these symptoms might indicate an undiagnosed serious pathology. In the CoBH vignette, only one participant explicitly explained the possibility of bowel cancer when safety‐netting; others simply advised patients to represent if they noticed any blood in their stool or any weight loss. Similarly, in the headache vignette, just one participant was explicit about the possibility of diagnostic error in their safety‐netting, explaining that it was important to reattend to make sure a serious diagnosis had not been missed. Conversely, 77% safety‐netted without mentioning this risk, simply telling the patient to return if symptoms worsened/did not resolve.

### 3.2 What were the reasons for the communication behaviours observed?

The semi‐structured interviews explored why participants chose to (not) communicate diagnostic uncertainty. We identified several themes: the impact of communication on patient anxiety and over‐investigation, communication as a way of building trust, protecting against diagnostic error and avoiding overwhelming patients.

#### 3.2.1 Patient anxiety

Avoiding patient anxiety was a common motivation for why participants communicated as they did, but they varied in how they thought this was best achieved. The majority felt that communicating diagnostic uncertainty—for example, mentioning unlikely but potentially serious diagnoses—could increase worry.

[W]hen you feel like it's not the most likely diagnosis … sometimes you don't want to scare the patient unnecessarily. (308)

This was often associated with cancer, which some reflected can be particularly anxiety‐provoking.

[C]ancers just cause huge anxiety to people whereas people … seem to be less worried about non‐malignant diagnosis, like angina, even though…they might have similar ramifications. (101)

Throughout our interviews, the use of euphemisms to avoid inducing patient anxiety was common:

I think just saying ‘something sinister’… it introduces the idea without planting horrible thoughts. (203)

For many, the balance between managing patient anxiety and communicating honestly was determined by the level of clinical suspicion surrounding the ‘sinister’ diagnosis. When the ‘sinister’ diagnosis was considered very unlikely, participants might not mention it.

I think until I've got a reasonable index of suspicion, I tend not to mention cancer just because it's a word that just carries so much anxiety with it. (101)

In contrast, a small number of participants felt that explicitly discussing the full differential diagnosis might *reduce* patient anxiety. Patients often already have concerns about serious diagnoses; not explicitly mentioning the full differential diagnosis might leave patients concerned that these conditions had not been properly considered, thus increasing anxiety.

[I]f the patient does go home and then sees their family and they say, oh but did they think it was cancer? And they say, I don't know. There's that sort of—did the doctor not consider it,'cause I didn't ask about it? So I introduced it that way. (202)

[M]y consulting style is to be very open with my thought process. I tend to explain exactly how I'm working things through to patients. And I think that reassures them that I've thought about it and not disregarded it. Because even if the patient says there's nothing on their mind, they might be too scared to say, ‘I'm worried about cancer’. (216)

Similarly, some acknowledged that patients can easily research their symptoms online. As such, patients are often already aware of concerning differentials that were being considered.

[I]f you just tell them the CT's normal, off you go … they might have read about it and might be worried about a bleed … I just think you should try and explain your reasoning to them. (206)

#### 3.2.2 Building trust and demonstrating competence

Many considered the impact that communication might have on the doctor–patient relationship, with most recognising the need to build trust and instil confidence; again, while the motivations were similar, the means varied.

Some suggested that open and honest communication about uncertainty was important in building trust.

I think you need to be open and show logical thinking, I think that helps build trust … it shows that you're making the effort to explain to them and educate them about their own body. (216)

Others felt that discussing uncertainty might make the patient doubtful of their competence.

[O]ne of the hardest things, as a doctor, is to try and convey to a patient [is] that … you're not sure you know what's going on with them—trying to get that across and maintain their confidence in you, especially if you say, ‘I've no idea what's wrong you’. (302)

One participant justified not discussing the risk of a false negative from the CT scan by referring directly to the need to maintain patient confidence:

There's no point sending someone home and then saying, oh, but it might have been a false negative, and just send them home with worry; that doesn't make sense. You need to be confident, and that confidence goes on to your patients. (303)

In contrast, others felt that *not* disclosing information about uncertainty might result in reduced patient trust/confidence in them.

[I]f further down the line it ends up that he does have cancer, it shows that you've thought about it and disregarded it in a logical way. It's not that you've not thought about it at all. And I think if they thought you'd disregarded it, that would make them lose confidence in you. (216)

#### 3.2.3 Avoiding diagnostic errors by empowering reattendance

Explicit discussions about diagnostic uncertainty as part of the safety‐netting process were relatively uncommon. Those who did discuss uncertainty often did so to encourage patients to voice concerns or reattend if symptoms worsened/persisted.

I think it is important sometimes to be transparent with patients and not to appear dismissive so I do like to explain to them that sometimes, you know, we get things wrong or we can't pick things up in the first instance and so if there is a problem, we're here to listen and they can come back with it. (101)

Discussing uncertainty was used to encourage patients to appropriately monitor their symptoms and understand their potential gravity.

[N]othing is 100 per cent accurate when it comes to medicine … So I think it's very important to say to the patients about the most serious conditions that you could have missed … so that in case we did miss something and the disease starts progressing or they start getting serious symptoms, then they can come in and get it checked again … I think safety‐netting is very, very important because of diagnostic uncertainty. (315)

Several participants explained that explicitly naming cancer as a possible diagnosis helped ensure patient vigilance with follow‐up, particularly for patients they perceived to be less likely to attend future appointments. Naming sinister differentials was believed to bolster patient self‐advocacy and mitigate against system weaknesses, which may cause patients to get ‘lost to follow up’.

I know the NHS can be slightly chaotic at times … so, if you say, we're doing this for cancer, then hopefully, if they don't hear about the results, or they don't get a follow‐up clinic … then they will phone up and go, what's going on with my cancer test results, as opposed to it just falling off the stack. (313)

#### 3.2.4 Concerns about overwhelming or overburdening patients

Many participants felt certain information might be too complex for patients: they did not want to overload patients. Some felt a duty to filter the medical jargon.

I suppose we should discuss pros and cons always in every investigation … but I worry I might be overburdening a patient with potentially medical jargon if I start talking about false negative and false positives. (304)

For the anaemia vignette, some participants did not name myeloma as a potential diagnosis because they suspected most patients would not have heard of it, or that there were too many differentials to list.

[M]yeloma is quite a difficult condition to explain to patients and they would perceive it as a cancer in the same way, so … I didn't think that the information was going to help her at that point. (302)

I think it would be slightly overwhelming to the patient to start listing … the huge number of differentials of a 75‐year‐old presenting with anaemia. (204)

#### 3.2.5 Desire to avoid unnecessary investigation

Many participants referred to the importance of making judicial use of investigations, acknowledging the potential harms of over‐investigation:

So we could do a CT scan on everybody to find anything, but there's a difference between medical decision‐making and diagnostics, making informed decisions to do the right test rather than actually doing a test on everyone. (305)

Some voiced concerns that by explicitly discussing diagnostic uncertainty they might induce patient desire for further (perhaps inappropriate) investigation.

[I]f you start talking about false negatives, then you invite the patient to almost question everything that you're doing and you enter this whole defensive mindset of having to do everything for every single patient. In science, there's an acceptable level of error that we're allowed… (303)

In contrast, one participant speculated that openly discussing uncertainty might facilitate better shared‐decision making, helping the patient to better understand that further tests are not required:

It might be they're very anxious, they might think that they've got something serious and panic … I'd say, look, it's not going to add anything … Every investigation has risks and benefits. (206)

For this participant, discussing their thought processes—including any ongoing diagnostic uncertainty—was a way of engaging with the patient and coming to an agreement about not doing further investigations.

#### 3.2.6 The patient‐specific nature of communication

Many discussed how they would try to tailor their communication to individual patients. Specifically, they would try to gauge a patient's anxiety level and determine their specific concerns before deciding how to communicate about diagnostic uncertainty.

I think if … they were particularly anxious that we might have missed something, then I would explain the … rationale for stopping the investigations. (101)

Other participants took the opposite approach, stating that for more anxious patients they might be less likely to mention certain concerning possible diagnoses.

I would judge it in reality based on the personality of the patient. I think if that explaining in that much detail is going to cause excess worry I don't think it's always beneficial. (310)

Beyond gauging anxiety levels, a few participants suggested that they might make judgements based on patient characteristics, such as age or cognitive status.

And I just think with the elderly, they do prefer just to hear it straight, and quite often they respond better. Whereas a younger patient might get very worried straightaway … the elderly patients have been through the healthcare system and they do understand it. (210)

#### 3.2.7 Training in communicating diagnostic uncertainty

Most reported that they had not received formal teaching on communicating diagnostic uncertainty. Several reflected that medical school teaching tends to emphasise *certainty*, in contrast to the realities of medical practice.

I don't think during medical school you are taught to deal with uncertainty. I think most of the … communication skills you're taught to deliver the diagnosis and explain the management and the treatment plan … when you come into actual practice, you realise sometimes you don't know the diagnosis. (209)

‘On the job’ learning was an important theme: both from observing seniors and through personal trial‐and‐error. The majority felt that more specific teaching on communicating diagnostic uncertainty would be helpful.

I think that is probably something that's missing [in current teaching] because it is something that we deal with every day … I think it is a really key thing that probably should be covered in communication teaching. (210)

## 4 DISCUSSION

### 4.1 Summary of main findings

We found variations in how doctors communicate diagnostic uncertainty to patients in a controlled setting. Although all participants were presented with identical clinical information, the manner and extent to which they communicated different aspects of diagnostic uncertainty varied significantly. Most of our participants described limited teaching in communicating diagnostic uncertainty, which may also have contributed to the variation observed.

While most participants explained investigation results and offered reassurance, we found differences in how they discussed differential diagnoses, how much they acknowledged their level of certainty in diagnoses/investigation results and whether they discussed uncertainty when safety‐netting. Implicit diagnostic uncertainty communication was more common than explicit. A range of considerations influenced doctors' communication choices: the potential impact on patient anxiety and the doctor–patient relationship; the complexity of information; a desire to avoid further unnecessary investigations and ideas about empowering patients to voice concerns or reattend if symptoms worsened.

Several factors are important in understanding the variation that we observed: (1) the varying—and often conflicting—communication goals that different participants expressed (and differences in how their underlying ethical principles were balanced), and (2) how different participants felt these goals could be achieved.

### 4.2 Conflicting ethical and communication goals

According to the multiple goals theory, high‐quality communication involves a successful balancing of multiple and sometimes conflicting goals.^40^ This framework has been applied to healthcare to examine communication in end‐of‐life care^41^ and paediatrics.^42^ In the latter study, paediatricians attended to multiple goals when communicating with parents about uncertainty. Our participants demonstrated several similar communication goals, including *task* goals (educating patients about what symptoms to look for and when to reattend), *relational* goals (providing reassurance and reducing anxiety) and *identity* goals (demonstrating their credibility through medical knowledge).

These communication goals can conflict with each other: for example, doctors must balance providing information about the possibility of a missed or misdiagnosis , at the same time providing reassurance; they must balance the need to openly acknowledge when there is diagnostic uncertainty with a need to maintain professional credibility and patient confidence.

Many of these competing communication goals can be framed in terms of conflicting underlying ethical principles. Similar tensions have been identified in debates surrounding therapeutic privilege and nondisclosures in clinical practice, in which consideration is given to how doctors weigh up avoiding harm (by disclosing distressing information) versus respecting autonomy (by providing patients with information to support them in making health decisions).^25^, ^26^, ^43^, ^44^

Although our participants rarely explicitly linked their communication goals to underlying guiding ethical principles, the goals they referred to can be mapped onto them. For example, many of our participants' motivations can be grounded in the ‘four pillars’ of medical ethics: autonomy, beneficence, nonmaleficence and justice.^22^ A majority of our participants expressed a desire to reduce patient anxiety, often born out of a desire to avoid causing harm to patients (nonmaleficence). In contrast, and consistent with other research,^45^, ^46^ other participants emphasised the importance of using communication to empower patients and enhance their agency, prioritising respect for autonomy.

Inherent to principlism as conceived by Beauchamp and Childress is pluralism: there is not a single overarching principle but a list of four moral principles. When the principles conflict, each must be weighed and balanced against each other in a process of reflective equilibrium. For some, the lack of clear, practically applicable guidance on how to balance competing principles is a serious flaw, as the principles often conflict in an unresolvable manner, resulting in disagreements and contradictions.^47^, ^48^

The fact that there is little specific guidance on how to practically balance the principles within Beauchamp and Childress' framework might explain the variation in how different participants in our study chose to value and prioritise distinct communication goals. Participants expressed different communication goals, and differences in how they balanced them in the context of conflicting ethical principles may explain some of the observed differences in communication.

### 4.3 Differing perspectives on how to achieve communication goals

The variation in communication we observed may also be attributable to differing perspectives on how to achieve the identified communication goals. Our participants expressed conflicting views about the impact of communicating diagnostic uncertainty to patients. Some felt that it would increase patient worry, while others felt that it could alleviate it; some felt that it would enhance patient trust/confidence, while others felt it would impair it and finally, some felt that communicating diagnostic uncertainty might drive over‐investigation, while others felt that such communication might improve shared understanding with patients about why further tests are not required. So, although some doctors had similar communication *goals*, different ideas about how to achieve them resulted in varying communication *behaviours*.

That the doctors in our study expressed different ideas about the impact of communicating diagnostic uncertainty on patients is perhaps unsurprising: many of the recommendations on how to communicate uncertainty lack an evidence base,^20^ and there is a paucity of patient‐focused research in this area.^28^, ^29^ Although there is evidence that communicating other aspects of uncertainty (in prognosis or treatment options) can negatively impact patient satisfaction,^13^, ^49^, ^50^, ^51^ little research directly examines the effects of communicating *diagnostic* uncertainty to patients. Existing studies identify conflicting trends. For example, a study in paediatrics found that explicit expression of diagnostic uncertainty was associated with lower perceived competence, and less trust and confidence.^52^ In contrast, a study involving patients with endometriosis found that they preferred doctors to share diagnostic uncertainty with them, to facilitate more informed decision‐making.^53^

The degree to which communicating diagnostic uncertainty might help prevent diagnostic error is also unclear. Doctors routinely ‘safety‐net’ (tell the patient which symptoms to look for and seek help for), and several authors suggest that communicating diagnostic uncertainty is an important as part of safety‐netting.^54^, ^55^, ^56^, ^57^ There is, however, little evidence that communicating uncertainty when safety‐netting actually reduces the incidence of diagnostic errors.^55^ In our study, some participants described sharing diagnostic uncertainty to protect against diagnostic errors by empowering patients to reattend; despite such expressed communication goals, explicit communication of diagnostic uncertainty when safety‐netting was very uncommon.

Overall, we identified that participants had conflicting opinions on what the impact of communicating uncertainty to their patients might be, and these conflicting opinions may explain some of the observed variations in communication. The lack of empirical evidence about the effects of communicating diagnostic uncertainty to patients makes drawing conclusions about the best approach challenging. There is a clear need for patient‐focused empirical research to establish how diagnostic uncertainty communication might impact upon outcomes (including patient anxiety, patient trust, resource utilisation and effectiveness of safety‐netting), as well as to better understand patient preferences.

### 4.4 Implications of variation in practice

The variation in communication that we observed itself has ethical implications. Our data suggest that different doctors may take very different approaches to the disclosure of information within the diagnostic process, which introduces the potential for considerable inequality between patients.

Although we asked participants to imagine they were conversing with a ‘typical patient’, in the follow‐up interview some participants alluded to how they would often be guided by the characteristics of the patient themselves in deciding what to communicate. While this is often considered (and can be) positive for patients, there is potential for it to accentuate inequalities, particularly in acute settings where there is not a pre‐existing therapeutic relationship. For instance, doctors may base their assumptions about what patients want to be told on characteristics, such as age, ethnicity or perceived education level, which have not been shown to accurately predict informational preferences. As one review of informational preferences in oncology concluded, ‘demographics do not reliably predict individual informational preferences, and studies have found contradicting results’.^58^

Overall, although communication should always be tailored to the individual patient, there is likely to be a benefit in some standardisation of diagnostic uncertainty communication. The current variation in communication practice is neither evidence‐based nor built upon compelling ethical analyses. Developing recommendations to guide how diagnostic uncertainty should be communicated may help reduce the influence of a clinician's unconscious biases in the provision of health information and enable a more equitable service.

### 4.5 Strength and limitations

This study permitted examination of communication in a controlled setting: using standardised vignettes, we compared participants' communication in response to the same clinical information. Using four common but diverse clinical scenarios increased the generalisability of our findings. All participants reported that they found the vignettes realistic, and all stated that they regularly see such patients in their clinical practice, suggesting external validity. Participants were recruited from a range of geographical locations and were evenly distributed across different grades (from first‐year doctors to consultants).

Although we took steps to make the scenarios as realistic as possible, the study is limited and the data collected may not necessarily reflect what really happens in clinical practice. There may be differences between what participants communicated in this controlled setting and what they actually communicated in real consultations (e.g. due to social desirability bias, where participants present an idealised version of their normal communication). The study design precluded any dialogue between the patient and the doctor. Real consultations involve conversation; we acknowledge that the absence of dialogue may have influenced results. This is salient given that many participants commented that their communication is often guided by the patient in front of them. It is also important to note that the observed behaviours were influenced by clinical judgement about the likelihood of certain diagnoses, as well as conscious reasoning about communication.

Finally, the quantitative data we present above (the code counts) should be interpreted with caution—our study was powered largely based on data saturation for the semi‐structured interviews discussing reasons for communication behaviours, so the sample size is relatively small.

## 5 CONCLUSION

This study develops the literature by specifically demonstrating that *diagnostic* uncertainty is communicated differently by different doctors, even when they are presented with identical clinical information in a controlled setting. We build on research that has demonstrated variability in approaches to uncertainty communication by confirming its presence in a UK secondary care context. This finding is particularly relevant considering both General Medical Council guidance and recent case law.

We highlight that doctors may have differing—and at times conflicting—communication goals, often reflecting conflicting ethical principles. Moreover, although doctors sometimes have similar communication goals, they often have differing opinions on how to achieve them. A combination of these factors, in addition to a lack of standardised training in diagnostic uncertainty communication, may underlie the variability in communication behaviours that we observed.

This study highlights the need for more research on patient perspectives. Such research may provide clarity by establishing what the impact of communicating diagnostic uncertainty is on patients, and by establishing what patients themselves value most, to help guide the balancing of different communication goals. This may help to inform recommendations, contributing to ongoing ethical and legal discussions about what obligations a doctor might have to disclose information relating to uncertainty in the diagnostic process.

## AUTHOR CONTRIBUTIONS

**Caitríona Cox**: Conceptualisation; methodology; investigation; formal analysis; writing—original draft; writing—review and editing; visualisation; data curation; project administration. **Thea Hatfield**: Investigation; formal analysis; writing—review and editing; data curation. **Zoë Fritz**: Conceptualisation; funding acquisition; writing—review and editing; supervision; methodology; project administration.

## CONFLICT OF INTEREST STATEMENT

The authors declare no conflicts of interest.

## ETHICS STATEMENT

The views expressed in this article are those of the authors and not necessarily those of the NHS, the NIHR or the Wellcome Trust. The study received approval from the University of Cambridge Psychology Research Ethics Committee. The participants of this study did not give written consent for their data to be shared publicly, only that the anonymised data collected may be used to support other researchers in the future. As such, the data that support the findings of this study are only available on request from the corresponding author, Caitríona Cox.

## Supporting information

Supporting information.

Supporting information.

Supporting information.

## Data Availability

The data that support the findings of this study are available from the corresponding author upon reasonable request.
