# Supplementary material for: How and why do doctors communicate diagnostic uncertainty: An experimental vignette study
Source: Health Expect. 2024 Jan 9;27(1):e13957. doi: 10.1111/hex.13957 (PMC10774830; doi:10.1111/hex.13957)
Supplement: Supplementary file 1 — Supporting information. [file HEX-27-e13957-s001.docx]

## **Appendix A: Semi-structure interview guide**

1. What were your reasons for discussing/not discussing the risk of missing a cancer diagnosis in vignette one, (for example when you safety-netted)?
2. What were your reasons for discussing/not discussing myeloma as a differential diagnosis in vignette 2?
3. In vignette 3, what were your reasons for discussing/not discussing the possibility that the pain could still be cardiac in nature, for example caused by angina?
4. What were your reasons for discussing/not discussing the possibility of the CT scan giving a false negative, and missing a subarachnoid haemorrhage? Why did you/did you not discuss your reasons for not doing an LP?
5. To what extent do you see patients like those presented in the above vignettes in your clinical practice?
6. Is there anything else you would like to tell us about communicating diagnostic uncertainty?
