# Supplementary material for: How and why do doctors communicate diagnostic uncertainty: An experimental vignette study
Source: Health Expect. 2024 Jan 9;27(1):e13957. doi: 10.1111/hex.13957 (PMC10774830; doi:10.1111/hex.13957)
Supplement: Supplementary file 3 — Supporting information. [file HEX-27-e13957-s003.docx]

**Appendix C: coding framework**

| Name | Description |
| --- | --- |
| **Vignette 1 codes IBS** |  |
| V1.1 explains normal Ix results | Any time doctor explains or mentions a test result. For example, 'Your blood results are normal'. Data can go into this code whether or not the doctor follows this with a statement of reassurance (e.g. 'All the tests we've done have been normal, which is reassuring'). |
| V1.2 Explicit reassurance about Ix or clinical features | Any time the doctor explicitly provides reassurance, either in relation to the test results or the clinical features (e.g. 'the results are very reassuring', 'there are no red flags', 'there is nothing that's making me worried'. |
| V1.3 discusses concept of ruling out serious or sinister causes | Doctor explains that we have done tests to 'rule out' or 'not miss' something serious or sinister. Data can be coded here even if the doctor does not actually explain what these serious/sinister pathologies might be. Data here might include doctor explaining that our approach to organising tests is to look for the most serious or life-threatening things first. |
| V1.4 explicit discussion of the ddx | Doctor specifically names a condition they had considered as part of the ddx (including explaining that they have ruled this out). e.g. 'We sometimes worry about bowel cancer when people have had a change in bowel habit', or 'I think this is unlikely to be IBD'). ---> this code will overlap with v1.5, but it is possible that some participants will mention ddx without saying the level of certainty (e.g. 'We've ruled out IBD') |
| V1.5 explicit level of certainty in negative dx | Doctor explicitly describes their level of uncertainty regarding a dx they have ruled out. e.g. 'I am confident this is not x', 'It is very unlikely to be x', or 'The probability of this being x is very low'. This may or may not involve the doctor explaining the reason for their level of certainty. NB: do not include this if the doctor just says, 'I don't think this is x' - as they have not indicated how sure they are. |
| V1.6 Explicitly acknowledges working dx may be wrong | Explicit acknowledgement that the working dx (i.e. IBS) may be wrong. e.g. 'There is a small chance that we are missing something more serious such as cancer', or 'without a camera test I cannot say for 100% sure that you don't have cancer'. This could include if it's done as part of safety-netting. |
| V1.7 Explains dx of IBS | Explains working dx of IBS, including explanation of what IBS is |
| V1.8 explicit level of certainty in IBS | Doctor explicitly describes level of certainty around working dx of IBS, e.g. 'The most likely cause of your symptoms is IBS', or 'I am confident that this is IBS' |
| V1.9 safety nets without explicit acknowledgement of risk of dx error | Doctor safety nets (i.e. tells patient to come back if symptoms change or worsen) but does not explicitly explain why. e.g. no explanation that if symptoms worsen this might be a sign that the dx of IBS is incorrect, and there is a more serious underlying pathology. |
| V1.10 safety-nets with explicit acknowledgement of risk of dx error | Doctor safety nets (i.e. tells patient to come back if symptoms change or worsen) with explicit explanation as to why. e.g. if symptoms worsen this might be a sign that the dx of IBS is incorrect, and there is a more serious underlying pathology which needs to be investigated/treated. |
| V1.11 Asks patient for feedback to help guide communication | Doctor asks patient/mentions they would ask patient for feedback to guide communication e.g. 'How much would you like to be told about the tests?' or 'Are you someone who likes to have everything explained to them, or would you prefer for me to just get on with it?'. Can also include if the participant explains to the interviewer that they would ask the patient for their concerns/be guided by what they ask about |
| V1.12 other |  |
|  |  |
| **Vignette 2 codes anaemia** |  |
| V2.1 explains blood tests show anaemia and or explains what anaemia is | Explains that bloods showed low Hb, explains what anaemia is, and/or explains that tiredness is likely due to anaemia |
| V2.2 Explains need to do further tests to work out the cause for anaemia | Explains that tests have not told us the cause for the anaemia, and that we need to do more investigations to work out cause |
| V2.3 Acknowledges many potential causes | Explains that there are many potential causes/wide ddx at this stage. Data may be coded here if doctor does not explicitly name and specific diagnoses (e.g. just states, 'there are lots of things that could be causing your anaemia'). Data can also be coded here if they list a few different conditions (e.g. this could be a vitamin deficiency, it could be iron deficiency, it could be a side-effect of your medication...) |
| V2.4 names a benign cause for anaemia | Gives example of a specific non-malignant condition (e.g. says 'iron deficiency', 'vitamin deficiency', or 'related to your underlying disease') |
| V2.5 names malignancy as a potential cause | Uses word 'myeloma', 'cancer', 'blood cancer' |
| V2.6 discusses concept of not wanting to miss or needing to rule out something 'serious' or 'sinister' | Doctor explains that we need to organise tests to 'rule out' or 'not miss' something serious or sinister. Data can be coded here even if the doctor does not actually explain what these serious/sinister pathologies might be. Data here might include doctor explaining that our approach to organising tests is to look for the most serious or life-threatening things first. |
| V2.7 Explicit reassurance | Any time the doctor explicitly provides reassurance. (e.g. 'I don't think is anything to worry about'). This could also include doctor stating it's likely to be a non-worrying cause for the anaemia (e.g. 'It's most likely that this is just related to the methotrexate you've been taking') |
| V2.8 provides some idea of the likelihood of a specific ddx | Doctor explicitly describes how likely or unlikely it is that they think it is that the patient has one of the ddx e.g. 'This is most likely to be x', 'Overall I think it's unlikely that this is caused by x'. |
| V2.9 Asks patient for feedback to help guide communication | Doctor asks patient/mentions they would ask patient for feedback to guide communication e.g. 'How much would you like to be told about the tests?' or 'Are you someone who likes to have everything explained to them, or would you prefer for me to just get on with it?'. Can also include if the participant explains to the interviewer that they would ask the patient for their concerns/be guided by what they ask about |
| V2.10 other |  |
|  |  |
| **Vignette 3 chest pain** |  |
| V3.1 Explains normal Ix results | Any time doctor explains or mentions a test result. For example, 'Your ECG is normal'. Data can go into this code whether or not the doctor follows this with a statement of reassurance (e.g. 'All the tests we've done have been normal, which is reassuring'). |
| V3.2 Provides explicit reassurance about Ix or clinical features | Any time the doctor explicitly provides reassurance, either in relation to the test results or the clinical features (e.g. 'the results are very reassuring', 'there are no red flags', 'there is nothing that's making me worried'.) |
| V3.3 explicit discussion about ddx | Doctor specifically names a condition they had considered as part of the ddx (including explaining that they have ruled this out). e.g. 'I do not think this is a heart attack', or 'It’s very unlikely that the heart is the cause of the pain''). ---> this code will overlap with v3.4, but it is possible that some participants will mention ddx without saying the level of certainty (e.g. 'We've ruled out a PE') |
| V3.4 Explicitly describes level of certainty in negative dx | Doctor explicitly describes their level of uncertainty regarding a dx they have ruled out. e.g. 'I am confident this is not x', 'It is very unlikely to be x', or 'The probability of this being x is very low'. This may or may not involve the doctor explaining the reason for their level of certainty. NB: do not include this if the doctor just says, 'I don't think this is x' - as they have not indicated how sure they are. |
| V3.5 Explicitly acknowledges pain could still be cardiac in nature | Doctor explains that we cannot completely rule out cardiac cause for pain (i.e. angina) |
| V3.6 Acknowledges cannot give definite cause for pain at moment | Explicitly states we cannot give definite cause for pain, or explains that we don't know cause for pain |
| V3.7 Names speculative benign diagnosis | Suggests a potential cause for pain (e.g. 'this could be a muscle strain') |
| V3.8 Explains concept of ruling out serious or sinister causes | Doctor explains that we have done tests to 'rule out' or 'not miss' something serious or sinister. Data can be coded here even if the doctor does not actually explain what these serious/sinister pathologies might be. Data here might include doctor explaining that our approach to organising tests is to look for the most serious or life-threatening things first. |
| V3.9 Safety nets without explicit discussion about the possibility this is still cardiac or dx error | Doctor safety nets (i.e. tells patient to come back if symptoms change or worsen) but does not explicitly explain why. e.g. no explanation that if symptoms worsen this might be a sign that the dx of non-cardiac chest pain is incorrect, and there is a more serious underlying pathology. |
| V3.10 Safety nets with explicit explanation that this could still be cardiac or dx error | Doctor safety nets (i.e. tells patient to come back if symptoms change or worsen) with explicit explanation as to why. e.g. if symptoms worsen this might be a sign that the dx of non-cardiac chest pain is incorrect, and there is a more serious underlying pathology which needs to be investigated/treated. |
| V3.11 Asks patient for feedback to guide communication | Doctor asks patient/mentions they would ask patient for feedback to guide communication e.g. 'How much would you like to be told about the tests?' or 'Are you someone who likes to have everything explained to them, or would you prefer for me to just get on with it?'. Can also include if the participant explains to the interviewer that they would ask the patient for their concerns/be guided by what they ask about |
| V3.12 other |  |
|  |  |
|  |  |
|  |  |
| **Vignette 4 headache** |  |
| V4.1 explains normal Ix results | Any time doctor explains or mentions a test result. For example, 'Your CT head is normal'. Data can go into this code whether or not the doctor follows this with a statement of reassurance (e.g. 'All the tests we've done have been normal, which is reassuring'). |
| V4.2 Provides explicit reassurance about Ix or clinical features | Any time the doctor explicitly provides reassurance, either in relation to the test results or the clinical features (e.g. 'the results are very reassuring', 'there are no red flags', 'there is nothing that's making me worried'.) |
| v4.3 Explicit discussion about ddx | Doctor specifically names a condition they had considered as part of the ddx (including explaining that they have ruled this out). e.g. 'We sometimes worry about a bleed on the brain in sudden headaches', or 'I think this is unlikely to be meningitis'). ---> this code will overlap with v4.4, but it is possible that some participants will mention ddx without saying the level of certainty (e.g. 'We've ruled out a brain tumour') |
| v4.4 Explicitly describes level of certainty in this negative dx | Doctor explicitly describes their level of uncertainty regarding a dx they have ruled out. e.g. 'I am confident this is not x', 'It is very unlikely to be x', or 'The probability of this being x is very low'. This may or may not involve the doctor explaining the reason for their level of certainty. NB: do not include this if the doctor just says, 'I don't think this is x' - as they have not indicated how sure they are. |
| v4.5 Discusses sensitivity of CT in some capacity | Discusses how sensitive the CT scan is for picking up SAH in some capacity (e.g. 'CT scans done within 6 hours are very good at picking up bleeds', or 'The scan we've done is very sensitive'). This can include use or numbers. It can also include if doctor explains CT scan will pick up 'almost all' but not every bleed |
| v4.6 Discusses concept of LP and or explains reason for not doing one here | Doctor mentions LP. This may include a brief explanation of not needing one here (e.g. we sometimes do an additional test called an LP, but in your case I don't think we need to'), or a more detailed explanation of why it is not being done. |
| v4.7 Explicitly acknowledges that we cannot 100% rule out a SAH | Doctor explains that we cannot 100% rule out SAH without an LP/explains there is still a tiny risk of missing a SAH |
| v4.8 Explains concept of ruling out serious or sinister causes today | Doctor explains that we have done tests to 'rule out' or 'not miss' something serious or sinister. Data can be coded here even if the doctor does not actually explain what these serious/sinister pathologies might be. Data here might include doctor explaining that our approach to organising tests is to look for the most serious or life-threatening things first |
| v4.9 Acknowledges that cannot give definite answer to what is causing the headache | Acknowledges that we don't know exactly what has caused headache, |
| v4.10 Names speculative benign diagnosis | Names specific condition which could be causing headache (e.g. 'This could just be a migraine'). |
| v4.11 Safety nets without explicit discussion about the possibility that this is a SAH | Doctor safety nets (i.e. tells patient to come back if symptoms change or worsen) but does not explicitly explain why. e.g. no explanation that if symptoms worsen this might be a sign that the there has been a diagnostic error and the headache is actually a SAH |
| v4.12 Safety nets with explicit explanation that this could be SAH | Doctor safety nets (i.e. tells patient to come back if symptoms change or worsen) with explicit explanation as to why. e.g. if symptoms worsen this might be a sign that the dx of non-SAH is incorrect, and there is a more serious underlying pathology which needs to be investigated/treated. (e.g. 'If the headache gets worse it's important that you come back, as there's a still very small chance that you have had a small SAH which wasn't picked up on the scan'). |
| V4.13 Asks patient for feedback to guide communication | Doctor asks patient/mentions they would ask patient for feedback to guide communication e.g. 'How much would you like to be told about the tests?' or 'Are you someone who likes to have everything explained to them, or would you prefer for me to just get on with it?'. Can also include if the participant explains to the interviewer that they would ask the patient for their concerns/be guided by what they ask about. |
| v4.14 other |  |
